# Metabolomic Biomarkers for the Human Diagnosis of Neglected Tropical Diseases: A Systematic Review

**DOI:** 10.1590/0037-8682-0298-2025

**Published:** 2026-08-07

**Authors:** Jéssica Burlamaque Maciel, Rebeca Linhares Abreu, Victor Irungu Mwangi, Camila Fabbri, Asenate Aline Xavier Adrião, Zeca Manuel Salimo, Michael Nosano Yakubu, Luíz Gustavo Araújo Gardinassi, Lúcia Helena Faccioli, Hector Henrique Ferreira Koolen, Gisely Cardoso de Melo, Marco Aurélio Sartim, Wuelton Marcelo Monteiro

**Affiliations:** 1 Fundação de Medicina Tropical Doutor Heitor Vieira Dourado, Departamento de Ensino e Pesquisa, Manaus, AM, Brasil.; 2 Universidade do Estado do Amazonas, Programa de Pós-Graduação em Medicina Tropical, Manaus, AM, Brasil.; 3 Fundação Oswaldo Cruz Amazônia, Instituto Leônidas e Maria Deane, Laboratório de Controle e Diagnóstico de Doenças Infecciosas, Manaus, AM, Brasil.; 4 Universidade Federal do Amazonas, Faculdade de Ciências Farmacêuticas, Manaus, AM, Brasil.; 5 Universidade do Estado do Amazonas, Escola Superior de Ciências das Saúde, Programa de Pós-Graduação em Biodiversidade e Biotecnologia da Rede BIONORTE, Manaus, AM, Brasil.; 6 AIDS Healthcare Foundation (AHF), Los Angeles, CA, USA.; 7 Federal University Wukari, Department of Microbiology, Wukari, Nigeria.; 8 Universidade de São Paulo, Escola de Enfermagem de Ribeirão Preto, Departamento de Enfermagem Materno-Infantil e Saúde Pública, Ribeirão Preto, SP, Brasil.; 9 Universidade de São Paulo, Faculdade de Ciências Farmacêuticas de Ribeirão Preto, Departamento de Análises Clínicas, Toxicológicas e Bromatológicas, Ribeirão Preto, SP, Brasil.; 10 Universidade do Estado do Amazonas, Escola Superior de Ciências das Saúde, Centro Multiusuário para Análise de Fenômenos Biomédicos (CMABio), Manaus, AM, Brasil.; 11 Duke Global Health Institute, Duke University, Durham, CN, USA.

**Keywords:** Metabolomics, Neglected tropical diseases, Human host metabolome, Human diagnosis

## Abstract

Neglected tropical diseases (NTDs) affect more than 1.6 billion people worldwide and remain major contributors to morbidity, disability, stigma, and poverty. Metabolomics has emerged as a promising approach for investigating disease-associated biochemical alterations and identifying biomarkers for diagnosis, prognosis, and treatment monitoring. This systematic review evaluated the available evidence on metabolomic profiles in WHO-defined NTDs and their potential clinical applications. MEDLINE/PubMed, Web of Science, and Scopus were searched through March 2026. Observational studies investigating metabolomic profiles in human subjects with NTDs were included, and methodological quality was assessed using a modified QUADOMICS tool. Seventy-four studies met the inclusion criteria. Most were conducted in China and Brazil and focused on dengue, onchocerciasis, schistosomiasis, leprosy, and Chagas disease. Across diseases, metabolomic alterations were predominantly observed in amino acid, lipid, carbohydrate, and energy metabolism pathways. Several candidate biomarkers were identified, including N-acetyltyramine-O-β-glucuronide for onchocerciasis, bile acid derivatives for schistosomiasis, amino acids and peptides for Chagas disease, lipid mediators for leprosy, and lipid- and serotonin-related metabolites for dengue. However, most studies remained exploratory, and only two validated biomarkers in independent populations. Risk-of-bias assessment revealed frequent methodological limitations, including inadequate reporting of pre-analytical procedures, insufficient characterization of study populations, and lack of strategies to address overfitting. Human metabolomics studies were absent for several WHO-defined NTDs, highlighting major research gaps. Future research should prioritize standardized methodologies, multicenter validation studies, and greater inclusion of underrepresented diseases and endemic populations to translate metabolomic discoveries into clinically useful tools for NTD diagnosis, monitoring, and control.

## INTRODUCTION

**FIGURE 1:**
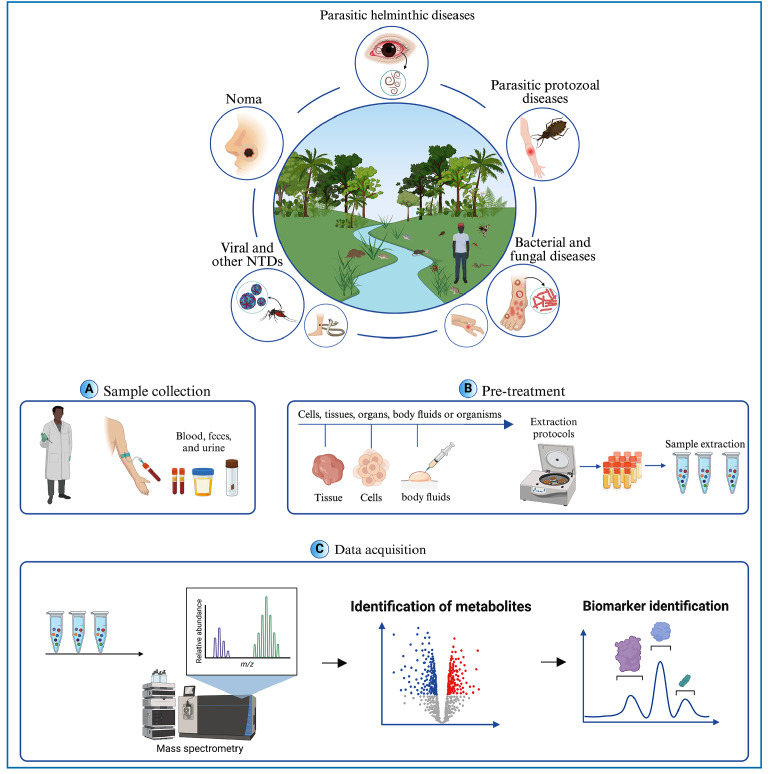
Neglected Tropical Diseases biomarker identification process from sample collection to metabolomic analysis. The figure was created in BioRender. **A)** The types of samples that can be used in metabolomics studies of NTDs, including blood, urine, and saliva. **B)** The pre-processing stage where collected samples are prepared for metabolite extraction. Depending on the type of sample (cells, tissues, or fluids), specific pre-treatment methods are applied, ensuring proper extraction of relevant compounds crucial for obtaining high-quality data. **C)** Data acquisition from processed samples using MS analysis. This and other analytical chemistry techniques enable the precise identification and quantification of metabolites. Finally, the resulting data is analyzed to identify disease-specific biomarkers based on differential abundance and database comparison which can be instrumental for diagnostics, monitoring, and advancing the development of new therapies for NTDs.

Neglected tropical diseases (NTDs) comprise a group of conditions that disproportionately affect impoverished populations in tropical and subtropical regions, where limited access to healthcare and sanitation sustains disease transmission and health inequities. These conditions include parasitic, fungal, bacterial, and viral diseases, as well as snakebite envenoming (Figure 1). According to the World Health Organization (WHO), more than 1.6 billion people require interventions for at least one NTD annually. NTDs contribute to disability, stigma, reduced quality of life, and persistent poverty, imposing substantial social and economic burdens^1^.

Advances in omics sciences have transformed biomedical research by enabling comprehensive characterization of biological systems. Among these approaches, metabolomics investigates small-molecule metabolites that reflect the combined influence of genetic, environmental, and pathological factors. Metabolites provide a sensitive snapshot of physiological and disease states and can reveal disease-associated biochemical signatures^2^. Metabolomics has been increasingly applied to infectious, cardiovascular, neurological, and neoplastic diseases, improving understanding of disease mechanisms, host-pathogen interactions, and treatment responses. By characterizing diverse metabolite classes, including amino acids, lipids, carbohydrates, and nucleotides, metabolomics has also emerged as a promising strategy for biomarker discovery, supporting the identification of diagnostic, prognostic, and treatment-monitoring indicators with potential applications in precision medicine^3^ ^-^ ^12^.

Evidence regarding the application of metabolomics to NTDs remains fragmented across diseases, analytical platforms, and clinical contexts. This systematic review aimed to assess the available evidence on metabolomic profiles in NTDs and evaluate their potential applications as biomarkers for diagnosis, disease monitoring, prognosis, and clinical management.

## METHODS

This systematic review was registered with PROSPERO (CRD420261377766) on May 8, 2026, and conducted and reported according to the PRISMA guidelines^13^. Two reviewers (JBM and RLAN) independently screened titles and abstracts for eligibility, while a third reviewer (CF) resolved discrepancies through consensus discussion. Full-text screening, methodological quality assessment, risk-of-bias evaluation using the QUADOMICS tool^14^, and data extraction were performed independently by JBM and WMM. Disagreements were resolved by consensus. 

### Literature Search

Search strategies were developed using MeSH terms and keywords related to metabolomics and WHO-defined NTDs. A draft MEDLINE search strategy is provided in Supplementary Table 1 and was adapted for Web of Science and Scopus. Searches covered studies published up to March 2026 and combined (“metabolomic” OR “metabolomics”) with (“neglected tropical diseases”, “NTDs”, the name of each NTD, or its etiological agent). Only articles published in English were included, with no geographical restrictions. Reference lists of all included studies were also screened for additional relevant publications. 

### Eligibility Criteria

We included observational studies (cohort, cross-sectional, and case-control) evaluating metabolomic profiles in patients with NTDs. Eligible studies investigated human subjects diagnosed with a WHO-defined NTD, analyzed human-derived biological samples (e.g., serum, plasma, urine, feces, or tissues) using targeted or untargeted metabolomics approaches, and were published in indexed scientific journals. Studies were required to employ established metabolomics platforms, including liquid chromatography-mass spectrometry (LC-MS), capillary electrophoresis-mass spectrometry (CE-MS), gas chromatography-mass spectrometry (GC-MS), or nuclear magnetic resonance (NMR), and to report metabolite-level data or candidate biomarkers. Studies were excluded if they did not investigate WHO-defined NTDs; were reviews, duplicate publications, conference abstracts, theses, dissertations, or preprints; did not use metabolomics as the primary analytical approach; or were restricted to *in vitro* or *in vivo* experimental models, animal reservoirs, vectors, non-human samples, or isolated etiological agents. 

### Assessment of Risk of Bias

Methodological quality and risk of bias were assessed using a modified version of the QUADOMICS tool^14^, developed for omics-based studies and derived from the Quality Assessment of Diagnostic Accuracy Studies (QUADAS) framework^15^. Six domains were evaluated and classified as presenting low, high, or unclear risk of bias according to predefined criteria: (1) participant selection; (2) sample description; (3) sample collection procedures and timing; (4) pre-analytical procedures and specimen handling; (5) execution of metabolomics analyses; and (6) risk of overfitting. Risk of bias was classified as low, high, or unclear when information was insufficient. 

### Data Extraction

Data were extracted independently by two reviewers (JBM and VIM) using a standardized form, with disagreements resolved by consensus or consultation with a third reviewer (CF). Extracted information included study characteristics, participant demographics, biological samples, metabolomics methodologies, analytical platforms, metabolite identification strategies, statistical analyses, comparator groups, identified metabolites and metabolic pathways, diagnostic performance metrics (AUC - area under the curve, sensitivity, specificity, and accuracy), validation approaches, and overfitting assessments. Information regarding robustness, reproducibility, and clinical applicability, including validation in independent cohorts and feasibility of clinical implementation, was also recorded. When necessary, metabolite nomenclature and reported metrics were standardized to facilitate comparisons across studies. 

### Data Synthesis

Due to substantial heterogeneity across studies regarding NTDs, biological samples, metabolomics platforms, analytical methodologies, statistical approaches, and reported outcomes, quantitative synthesis was not considered appropriate. Therefore, findings were synthesized narratively and presented in text and tables. The synthesis focused on study characteristics, identified metabolites and metabolic pathways, methodological features, diagnostic performance metrics, and the clinical applicability of reported biomarkers. Methodological quality and risk-of-bias assessments obtained using the QUADOMICS tool^14^ were considered during data interpretation and discussed alongside the study findings.

## RESULTS

### Study Characteristics

**FIGURE 2:**
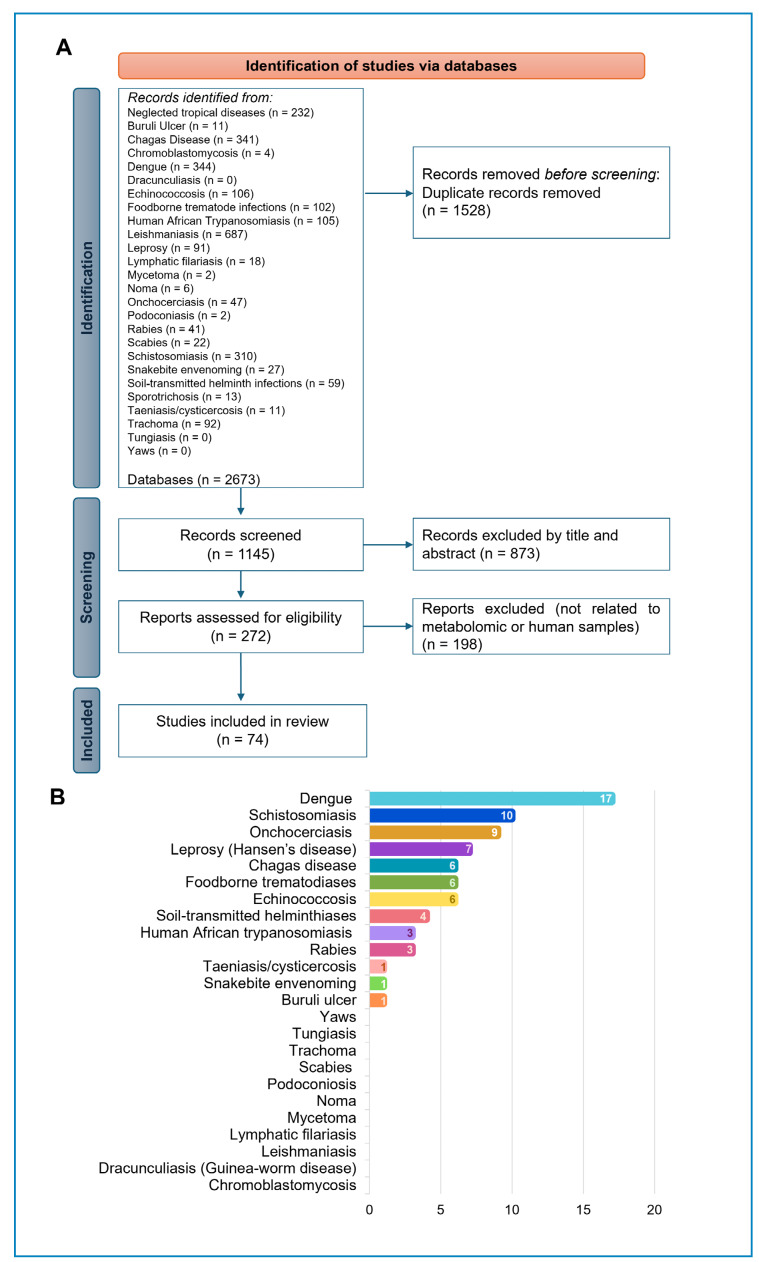
PRISMA flow diagram. **A)** PRISMA (Preferred Reporting Items for Systematic Reviews and Meta-Analyses) flow diagram detailing the study selection process. The diagram includes the number of records identified, screened, assessed for eligibility, and included in the systematic review. **B)** Graphical representation of the distribution of published articles on metabolomics across the various NTDs until March 2026.

Figure 2A summarizes the study identification and selection process. Our search strategy identified 2,673 citations. After removing duplicates and clearly ineligible records, 272 potentially eligible studies underwent full-text assessment, of which 198 were excluded according to the predefined criteria. Ultimately, 74 studies^16^ ^-^ ^89^ met the inclusion criteria, highlighting the limited application of metabolomics to NTDs.

**FIGURE 3:**
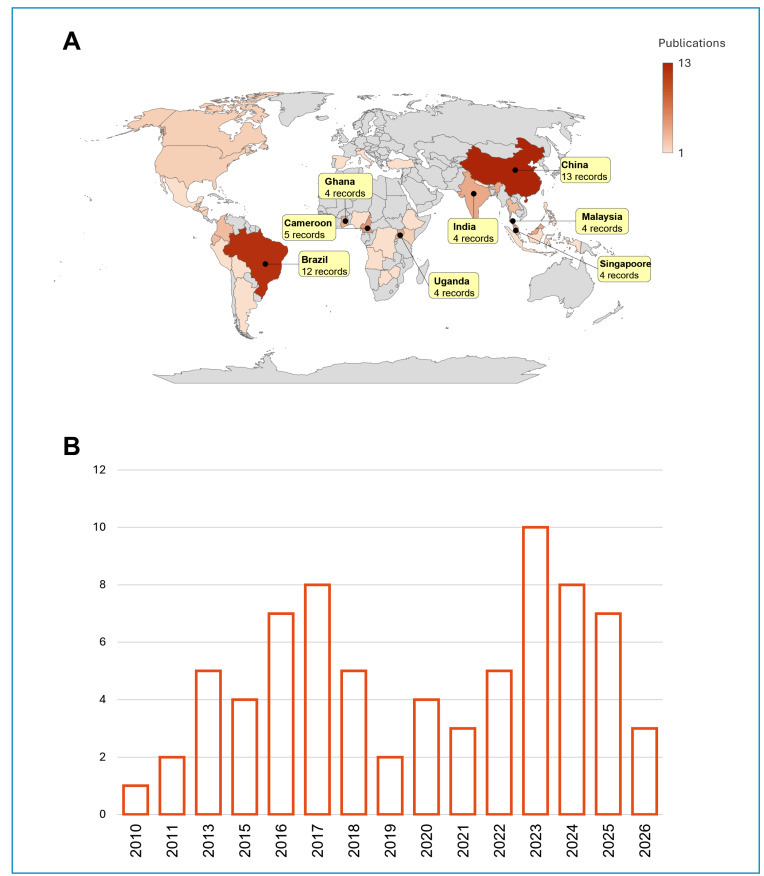
Distribution of studies on metabolomic biomarkers in neglected tropical diseases (NTDs) by country and year. **A)** Distribution of studies by country. **B)** Distribution of studies by year.

The main characteristics of the included studies reporting potential biomarkers are presented in Table 1. Figure 3A shows the geographical distribution of studies. Most were conducted in China (13 studies)^18^ ^-^ ^22^ ^,^ ^36^ ^,^ ^37^ ^,^ ^64^ ^,^ ^70^ ^,^ ^76^ ^,^ ^79^ ^,^ ^86^ ^,^ ^89^ and Brazil (12 studies)^38^ ^,^ ^39^ ^,^ ^43^ ^,^ ^45^ ^,^ ^46^ ^,^ ^55^ ^,^ ^56^ ^,^ ^71^ ^,^ ^77^ ^,^ ^83^ ^,^ ^87^ ^,^ ^88^, while the remaining studies were mainly carried out in Africa and Asia. Seven studies included populations from more than one country. Figure 3B shows the distribution of publication years. The included studies were published between 2010 and 2026, with the highest number of publications in 2023 (n=11).

**TABLE 1:** Main characteristics and findings of studies reporting potential biomarkers for neglected tropical diseases included in the systematic review.

NTD; Agent	First author, Year, Country	Biological sample	Case definition (n)	Control definition (n)	Mean age; male (%)	Analytical platform; metabolomics approach	Potential biomarkers; AUC	Biomarker type
Echinococcosis; *Echinococcus granulosus*	Ciftci, 2021^16^, Turkey	Plasma	Echinococcosis patients (36 active and 17 inactive cysts) (n=53)	Healthy controls (n=31)	Cases: 38.1, 39.6%; Control: 37.4, 48.4%	GC-MS and LC-MS; Untargeted	Squalene (AUC=0.869), 3-phosphoglycerate (AUC=0.848), glutamic acid (AUC=0.841), glyceric acid (AUC=0.805), palmitoleic acid (AUC=0.804) and oleic acid (AUC= 0.780)	Diagnostic biomarker
Echinococcosis; *E. granulosus and E. multilocularis*	Bai, 2023^18^, China	Serum	Echinococcosis patients (14 alveolar echinococcosis and 16 cystic echinococcosis) (n=30)	Healthy controls (n=15)	Cases: AE 27.9, 40%/CEC: 28.4, 45%/Control: 28.2, 42.5%	GC-MS; Untargeted	2-hydroxyl-3-isopropylbutanedioic acid (AUC=0.752), 3-hydroxyl-3-methylglutaric acid (AUC=0.848), α-santonin (AUC=0.824), androsterone (AUC=0.771), hydroxyurea (AUC=0.702), and lactate (AUC=0.826)	Diagnostic biomarker
Ecchinococcosis; *E. multilocularis*	Aimati, 2025^19^, China	Serum	Hepatic cystic echinococcosis patients (n=10)	Healthy controls (n=13)	Cases: 36.2, 60%; Control: 37.9, NR.	LC-MS; Untargeted	Isopropamide (AUC of 0.82), Sphinganine (AUC=0.81), 15(S)-HETE-d8 (AUC=0.78), Allantoic acid (AUC=0.75), Tyr-Arg-Leu (AUC=0.72)	Diagnostic biomarker
Ecchinococcosis; *E. multilocularis*	Lin, 2019^20^, China	Serum and urine	Hepatic alveolar echinococcosis patients (n=18)	Healthy controls (n=18)	Cases: 32, 50%; Control: 34, 66.7%	¹H-NMR spectroscopy; Untargeted.	Serum: glutamate (AUC=0.924), leucine (AUC=0.920), valine (AUC=0.920) and phenylalanine (AUC=0.914), creatine (AUC=0.870), isoleucine (AUC=0.852), lysine (AUC=0.849), formate (AUC=0.833), betaine (AUC=0.824) and serine (AUC=0.824) (panel AUC=0.984). Urine: glutamate, creatinine and p-hydroxyphenylacetate (AUC=0.835)	Diagnostic biomarker
Onchocerciasis, *Onchocerca volvulus*	Globisch, 2013^27^, 2017^28^, Cameroon	Urine	Onchocerciasis positive samples (n = 145)	Onchocerciasis negative samples (n = 118)	NR	LC-MS; Targeted.	NATOG; NR	Diagnostic biomarker
Onchocerciasis, *Onchocerca volvulus*	Lagatie, 2021^29^, Ghana and Kenya	Plasma	Untargeted study: O. volvulus infected cases (n=68); Validation: Nodule-positive individuals (n=98).	Untargeted study: Non-endemic controls (n=20); lymphatic filariasis (n=8). Validation: Endemic controls (n=51); Non-endemic controls (n=54); Lymphatic filariasis (n=50); Healthy Belgian controls (n=50); Non-endemic controls, Kenya (n=476)	Nodule-positive, Ghana: 47; 53%. Non-endemic controls, Ghana: 23; 67%. Endemic controls, Ghana: 35; 51%. Lymphatic filariasis, Ghana: 33; 72%. Healthy controls, Belgium: 40.5; 44%. Non-endemic controls, Kenya: 11; 50%.	LC-MS; Untargeted and Targeted.	Hypoxanthine (AUC=0.93) and inosine (AUC=0.91)	Diagnostic biomarker
Onchocerciasis, *Onchocerca volvulus*	Wewer, 2021^32^, Cameroon	Urine	Ov-infected (n = 41); Ov + Ll (n=6); Ov + Mp (n=8); Ov + Ll + Mp (n=3). Total n = 58	Uninfected individuals (n=24); *Loa loa*-infected individuals (n=6); *Mansonella perstans*-infected individuals (n=5)	NR	LC-MS; Targeted.	NATOG and cis-cinnamoylglycine; NR.	Diagnostic biomarker
Schistosomiasis, *Schistosoma haematobium*	Adebayo, 2018^35^, Nigeria	Urine and plasma	Infected (n=65)	Healthy controls (n=46)	NR; 56.8%	LC-MS; Untargeted	Catechol estrogens (AUC=0.66-0.95)	Diagnostic biomarker
Schistosomiasis, *Schistosoma japonicum*	Li, 2024^36^, China	Serum	Chronic *S. japonicum* infection (n=33); Advanced schistosomiasis (n=15). Total n=65	Healthy controls (n=17)	CSJ: 58.2; 63.6%. ASJ: 64.4; 46.7%. Control: 57.8; 64.7%	LC-MS; Untargeted	Glycocholic acid (AUC=0.838), glycochenodeoxycholate (AUC=0.828) and taurochenodeoxycholic acid (AUC=0.027)	Disease staging biomarker
Soil-transmitted helminthiases; *Ascaris lumbricoides*	Lagatie, 2020^41^, Kenya, Indonesia, Ethiopia	Urine and plasma	Untargeted study: *A. lumbricoides* infected (n=38). Validation: *A. lumbricoides* positive (n=198)	Untargeted study: Endemic controls (n=118); Non-endemic controls (n=40). Validation: Endemic controls (n=857); Non-endemic controls (n=214).	NR	LC-MS; Untargeted and Targeted.	2-MPC (AUC=0.81)	Diagnostic/infection intensity/treatment monitoring biomarker
Soil-transmitted helminthiases; *Strongyloides stercoralis*	Montanhaur, 2022^43^, Brazil	Stool	Strongyloidiasis-positive (n=10)	Healthy controls (n=15)	Cases: 53.4; 70.0%. Control: 48.5; 33.3%.	DI-HRMS; Untargeted.	Caprylic acid, mannitol, glucose, lysophosphatidylinositol and hydroxy-dodecanoic acid (AUC=0.965)	Diagnostic biomarker
Chagas Disease, *Trypanosoma cruzi*	Herreros-Cabello, 2024^45^, Brazil, Bolivia, Ecuador, Honduras, Colombia	Serum	Indeterminate Chagas disease (ICD) (n=31); Chronic Chagasic cardiomyopathy (CCC) (n=41)	Other cardiomyopathies (HD) (n=18); Healthy volunteers (n=30)	ICD: 45,4; 45.2%. CCC: 52.3; 58.5%; HD: 56.1; 72.5%; Controls: 50.8; 56.7%.	LC-MS; Untargeted.	Phenylacetylglutamine, fibrinopeptide B (1-13), bilirubin, biliverdin, urobilin, cystathionine, phenol glucuronide and vanillactate. NR.	Disease staging biomarker
Chagas Disease, *Trypanosoma cruzi*	Ambrósio, 2024^48^, Argentina	Plasma	CCD patients (n=30);	Healthy controls (n=17).	Indeterminate: 49.61; 33.3% male; Mild CCC 50.14; 14.3%. Severe CCC 48.85; 28.6%. Control: 43.37; 29.4%.	LC-MS; Targeted.	N-acetylserotonin; NR.	Prognostic biomarker
Chagas Disease, *Trypanosoma cruzi*	Golizeh 2022^49^, Switzerland	Serum	Chagas Disease patients (n=24)	Healthy controls (n=24)	Cases: 43; 8%. Control: 39; 4%.	LC-MS; Untargeted.	Taurine, glutamine, pGlu-Gly, Phe-Thr, Asn-Gly-Phe-Lys; NR.	Diagnostic biomarker
Human African trypanosomiasis, *Trypanosoma brucei gambiense*	Vincent, 2016^55^, Angola	Cerebrospinal fluid	HAT stage 1 (n=20); HAT stage 2 (n=20)	Healthy controls (n=16)	HAT stage 1: 43; 50%. HAT stage 2: 35.5; 65%. Control: 35; 37.5%.	LC-MS; Untargeted.	Neopterin and 5-hydroxytryptophan; NR.	Disease staging biomarker
Leprosy (Hansen’s disease), *Mycobacterium leprae*	Silva, 2023^56^, Brazil	Serum	Household Contacts That Developed Leprosy (HCDL) (n=17)	Household Contacts That Did Not Develop Leprosy (HCNDL) (n=17)	HCDL: 37.1; 35.2%. HCNDL: 45.2; 41,1%.	LC-MS; Targeted.	RvD1 and RvD2, and PGD2; NR.	Prognostic biomarker
Dengue fever; Dengue virus (DENV)	Cui, 2016^67^, Singapore	Serum	Dengue patients - 60 DF patients and 56 DHF patients (n=116)	Healthy controls (n=24)	Cases: 36,7*; NR. Control: NR	LC-MS; Untargeted and targeted.	Serotonin (AUC=0.8) + IFN-y (AUC=0.92)	Disease staging/Severity biomarker
Dengue fever; Dengue virus (DENV)	Soma, 2025^74^, Nicaragua	Serum and plasma	Dengue Fever (DF) (n=185); Dengue Hemorrhagic Fever (DHF) (n=44); Dengue Shock Syndrome (DSS) (n=22)	Negative dengue (n=284)	All patients: 8.3; 52%.	LC-MS; Untargeted.	Panel: Leucyl-alanine, L-prolyl-proline, Valyl-leucine, LPC(18:3), Serotonin, Phenylalanylphenylalanine, Arachidonic acid, Inosine, LPC(22:6), Creatinine, Carnitine, Hypoxanthine, MG(16:0), Docosahexaenoic acid Glycodeoxycholic acid, Hippuric acid, LPE(22:6), L-Phenylalanine, Indolepropionic acid, Proline, L-Tryptophan, LPC(18:0) sn1, Alpha-linolenic acid, Propionylcarnitine, Indoleacetic acid, Gamma-linolenic acid, Linoleic acid, 2-Octenoylcarnitine. (AUC=NR)	Prognostic biomarker
Dengue fever; Dengue virus (DENV)	Voge, 2016^75^, Mexico and Nicaragua	Serum	Dengue patients - 42 DF/26 DHF (n=68)	Non-dengue febrile illness (n=33)	NR; DF: 20.79%/ DHF: 11.88%/ND: 15.84%	LC-MS; Untargeted and targeted.	α-linolenic acid, arachidonic acid and docosahexaenoic acid, LysoPC (16:0), LysoPC (18:1), 1,25-dihydroxyvitamin D3 (AUC: NR)	Disease staging/Severity biomarker
Dengue fever; Dengue virus (DENV)	Fierro, 2025^84^, Ecuador	Serum	Children and adolescents infected with DENV (n=25)	Healthy controls (n=15)	NR	LC-MS; Untargeted.	*m/z* 246.2655 (AUC=0.798)	Diagnostic biomarker
Dengue fever; Dengue virus (DENV)	Josyula, 2024^85^, India	Plasma	Dengue patients - without warning signs (n=10), with warning signs (n=10), and severe dengue (n=10) (n= 30)	Healthy controls (n= 10)	NR; 60%.	LC-MS; Untargeted.	Stage disease: 13-S-HODE, 8-HETE, 12-HETE, 3-hydroxylinoleylcarnitine, malonylcarnitine, PG (36:1), propionylcarnitine, L-carnitine, 2-octenoylcarnitine, agmatine, and quinolinic acid (AUC=1). Diagnosis: Shikimic acid, Ureidosuccinic acid, Propionylcarnitine and AlphaTocopherol (AUC=0.75)	Diagnostic and disease staging/Severity biomarker
Dengue fever; Dengue virus (DENV)	Liu, 2026^70^, China	Plasma	Dengue patients (n=41)	Healthy controls (n=23)	Case: 40.78; 43.9%. Control: 33.3; 47.8%.	LC-MS; Untargeted.	Trans-cinnamic acide (AUC=0.843), L-acetylcarnitine (AUC=0.824), SM(d17:1/17:0) (AUC=0.8), 1,2,4,5-Cyclohexanetetrol (AUC=0.971), 5- (hydroxymethyl)pyrrolidin-2-One (AUC=1.000), 1,2,3,4-tetrahydro-6- propanoylpyridine (AUC=1.000), 2-C-methyl-D-erythritol-4-phosphate (AUC=0.721), physalolactone (AUC=0.948), S-japonin (AUC=0.812) and 9-tridecynoic acid (AUC=0.999)	Diagnostic biomarker
Rabies; *Lyssavirus (RABV)*	O'Sullivan, 2013^61^, United States	Cerebrospinal fluid	Rabies patients (n=9) (34 CSF samples)	Control CSF (n=25)	Cases: 18.8; 77.8%. Control: age 5-25 years; NR	¹H-NMR spectroscopy; Untargeted.	Lactate, pyruvate, creatine, malate, succinate, 3-hydroxybutyrate, acetoacetate, acetone, 2-oxyglutarate, glycine, quinolinate, lysine, methionine, phenylalanine, ornithine, urea, choline, ethanolamine. (AUC: NR)	Disease staging biomarker

**AUC:** area under the curve; **AE:** alveolar echinococcosis; **ASJ:** acute *Schistosoma japonicum* infection; **CEC:** cystic echinococcosis; **CCC:** chronic Chagasic cardiomyopathy; **CCD:** chronic Chagas disease; **CSF:** cerebrospinal fluid; **CSJ:** Chronic *Schistosoma japonicum*; **DI-HRMS:** direct infusion high-resolution mass spectrometry; **DF:** dengue fever; **DHF:** dengue hemorrhagic fever; **GC-MS:** gas chromatography-mass spectrometry; **HAT:** human African trypanosomiasis; **HCDL:** household contacts that developed leprosy; **HCNDL:** household contacts that did not develop leprosy; **HD:** other cardiomyopathies; **ICD:** indeterminate Chagas disease; **LC-MS:** liquid chromatography-mass spectrometry; **Ll:** *Loa loa*; **LPC:** lysophosphatidylcholine; **LPE:** lysophosphatidylethanolamine; **LysoPC:** lysophosphatidylcholine; **MG:** monoglyceride; **Mp:** *Mansonella perstans*; **NATOG:** N-acetyltyramine-O-glucuronide; **NTD:** neglected tropical diseases; **NR:** not reported; **Ov:** *Onchocerca volvulus*; **PG:** prostaglandins; **PGD2:** prostaglandin D2; **Phe-Thr:** phenylalanine-threonine; **pGlu-Gly:** pyroglutamyl-glycine; **13-S-HODE:** 13-S-hydroxyoctadecadienoic acid; **8-HETE:** 8-hydroxyeicosatetraenoic acid; **12-HETE:** 12-hydroxyeicosatetraenoic acid; **RvD1:** resolvin D1; **RvD2:** resolvin D2; **Tyr-Arg-Leu:** tyrosine-arginine-leucine; **¹H-NMR:** proton nuclear magnetic resonance; **15(S)-HETE-d8:** 15(S)-hydroxyeicosatetraenoic acid-d8.

### Diseases

Considerable heterogeneity was observed in metabolomics research across WHO-defined NTDs. Dengue, onchocerciasis, schistosomiasis, leprosy, and Chagas disease accounted for the largest proportion of publications (Figure 2B). In contrast, no eligible studies were identified for leishmaniasis, chromoblastomycosis and other deep mycoses, mycetoma, yaws, trachoma, scabies and other ectoparasites, podoconiosis, lymphatic filariasis, dracunculiasis, or noma (Figure 2B).

A total of 39 studies addressed parasitic helminth diseases. For echinococcosis, most studies (n=4) were conducted in China^18^ ^-^ ^21^. Among the six included studies^16^ ^-^ ^21^, four identified potential diagnostic biomarkers^16^ ^,^ ^18^ ^-^ ^20^. Although six studies on foodborne trematode infections were included, none progressed to the identification of potential biomarkers. Regarding onchocerciasis, nine studies were included^26^ ^-^ ^34^, of which eight were conducted in African populations^26^ ^-^ ^32^ ^,^ ^34^, mainly from Cameroon (n=5). Four studies identified potential biomarkers^27^ ^,^ ^28^ ^,^ ^30^ ^,^ ^32^. Ten studies investigated schistosomiasis^35^ ^-^ ^40^ ^,^ ^80^ ^-^ ^82^ ^,^ ^88^, but only two reported potential biomarkers^35^ ^,^ ^36^. Study populations mainly originated from Africa^35^ ^,^ ^40^ ^,^ ^80^ ^-^ ^82^ (n=5), while three studies were conducted in Brazil^38^ ^,^ ^39^ ^,^ ^88^. Four studies evaluated soil-transmitted helminths, specifically *A. lumbricoides* ^41^, *S. stercoralis* ^42^ ^,^ ^43^ and *T. trichiura* ^89^; however, only two identified potential biomarkers.

For protozoan diseases, nine studies were included. Six investigated Chagas disease^45^ ^-^ ^49^ ^,^ ^65^, three of which identified biomarkers^45^ ^,^ ^48^ ^,^ ^49^. Most were conducted in populations from South and Central America. Three studies evaluated human African trypanosomiasis^50^ ^-^ ^52^; two were conducted in Uganda^50^ ^,^ ^51^ and one in Angola^52^, which was also the only study to identify disease-staging biomarkers.

Eight studies investigated bacterial diseases. Seven focused on leprosy^54^ ^-^ ^56^ ^,^ ^77^ ^-^ ^79^ ^,^ ^87^, with only one reporting prognostic biomarkers. Four were conducted in Brazil^55^ ^,^ ^56^ ^,^ ^77^ ^,^ ^87^ and three in Asian populations^54^ ^,^ ^78^ ^,^ ^79^.

A total of 20 studies investigated viral diseases. Dengue accounted for the highest number of publications, with 17 studies^57^ ^-^ ^60^ ^,^ ^66^ ^-^ ^75^ ^,^ ^83^ ^-^ ^85^. Twelve were conducted in Asia^57^ ^-^ ^60^ ^,^ ^66^ ^-^ ^70^ ^,^ ^72^ ^,^ ^73^ ^,^ ^85^ and five in Latin America^71^ ^,^ ^74^ ^,^ ^75^ ^,^ ^83^ ^,^ ^84^. Despite the larger number of studies, only six identified potential biomarkers^67^ ^,^ ^70^ ^,^ ^74^ ^,^ ^75^ ^,^ ^84^ ^,^ ^85^. Rabies was represented by three studies^61^ ^-^ ^63^, all involving predominantly North American populations, and only one identified disease-staging biomarkers^61^.

Taeniasis/cysticercosis^44^, Buruli ulcer^53^, and snakebite envenomation^64^ were each represented by a single study. 

### Quality Assessment

All studies that identified potential biomarkers included comparison groups. Healthy individuals were the most common controls^16^ ^,^ ^18^ ^-^ ^20^ ^,^ ^35^ ^,^ ^36^ ^,^ ^43^ ^,^ ^45^ ^,^ ^48^ ^,^ ^49^ ^,^ ^52^ ^,^ ^67^. Other comparison groups included endemic and non-endemic controls^29^ ^,^ ^41^, disease-negative individuals^27^ ^,^ ^28^ ^,^ ^32^ ^,^ ^75^, patients with other diseases^29^ ^,^ ^32^ ^,^ ^45^, and household or exposed but non-diseased contacts^56^.

The risk-of-bias assessment is summarized in Supplementary Table 2. No study presented a low risk of bias across all six domains. Only one study^70^ fulfilled at least five of the seven criteria for low risk of bias. Thirty studies (40.5%)^20^ ^,^ ^22^ ^,^ ^23^ ^,^ ^29^ ^,^ ^32^ ^,^ ^36^ ^-^ ^39^ ^,^ ^41^ ^,^ ^42^ ^,^ ^44^ ^,^ ^49^ ^,^ ^51^ ^,^ ^52^ ^,^ ^54^ ^,^ ^56^ ^,^ ^60^ ^,^ ^61^ ^,^ ^62^ ^,^ ^65^ ^-^ ^69^ ^,^ ^72^ ^,^ ^75^ ^,^ ^76^ ^,^ ^80^ ^,^ ^81^ met at least four criteria, whereas another 30 studies (40.5%)^17^ ^-^ ^19^ ^,^ ^21^ ^,^ ^24^ ^,^ ^25^ ^,^ ^30^ ^,^ ^35^ ^,^ ^40^ ^,^ ^43^ ^,^ ^47^ ^,^ ^48^ ^,^ ^50^ ^,^ ^53^ ^,^ ^55^ ^,^ ^57^ ^-^ ^59^ ^,^ ^71^ ^,^ ^74^ ^,^ ^78^ ^,^ ^79^ ^,^ ^82^ ^-^ ^89^ met three criteria. Thirteen studies^16^ ^,^ ^26^ ^-^ ^28^ ^,^ ^31^ ^,^ ^33^ ^,^ ^34^ ^,^ ^45^ ^,^ ^46^ ^,^ ^63^ ^,^ ^64^ ^,^ ^73^ ^,^ ^77^ presented five or six methodological shortcomings.

Lagatie et al.^29^ and Lagatie et al.^41^ were the only studies to validate biomarkers using independent sample sets from distinct populations. Lagatie et al.^29^ validated biomarkers in a validation cohort and a non-endemic Kenyan population, whereas Lagatie et al.^41^ used independent cohorts from Kenya, Indonesia, Ethiopia, and Belgium. These approaches reduced the likelihood of overfitting and strengthened reproducibility. However, neither study reported the anticoagulant used during sample collection, which may slightly limit reproducibility.

The main reasons for high risk of bias were inadequate reporting of clinical characteristics, insufficient description of sample handling and pre-analytical procedures, and the lack of validation using independent sample sets or other approaches to address overfitting.

## DISCUSSION

Despite the marked etiological diversity of NTDs, the metabolomic studies included in this review reveal remarkable convergence in the metabolic pathways affected. Alterations involving amino acid metabolism, lipid remodeling, energy metabolism, and inflammatory mediators were consistently reported across protozoan, helminth, bacterial, and viral infections. 

### Parasitic helminth diseases

Helminthiases represented the most heterogeneous group of NTDs evaluated, yet recurring metabolic patterns were consistently observed, including alterations in amino acids, lipids, energy metabolism, bile acids, and purines across echinococcosis, schistosomiasis, onchocerciasis, and intestinal helminthiases. Furthermore, changes in eicosanoids, sphingolipids, and fatty acids suggest that modulation of the host inflammatory response is a common feature of helminth infections.

***Echinococcosis:*** Reported biomarkers were mainly associated with amino acid, lipid, and energy metabolism. Several carbohydrates, lipids, and amino acids distinguished cystic echinococcosis (CEC) patients from healthy controls^16^. Urinary citrate^17^ and three plasma metabolites^¹⁶^ differentiated fertile from infertile cysts, suggesting utility for assessing cyst viability. Five metabolites have also been proposed as promising biomarkers for early diagnosis^18^. More recently, Aimaiti et al.^¹⁹^ identified isopropamide, sphinganine, 15(S)-HETE-d8 (hydroxyeicosatetraenoic acid), allantoic acid, and Tyr-Arg-Leu (tyrosine-arginine-leucine) as candidate biomarkers. Additional studies reported elevated aromatic amino acids and glutamate, reduced branched-chain amino acids and creatine²⁰, and alterations in linoleic and arachidonic acid metabolism²¹. Urinary succinate was also proposed as a marker distinguishing CEC patients from individuals without hydatid cysts^17^.

***Foodborne trematode infections*:** Although six studies were included, none identified validated diagnostic biomarkers. Nevertheless, metabolomics highlighted differentially expressed metabolite classes^22^ ^-^ ^24^ and informed future biomarker discovery efforts^25^. Bile metabolomics revealed reduced amino acids in *Clonorchis sinensis* infection^22^ and marked decreases in bile acids^86^, including CDCA (chenodeoxycholic acid), UDCA (ursodeoxycholic acid), HDCA (hyodeoxycholic acid), and βCA (3β-cholic acid), although the invasive nature of bile collection limits clinical applicability. Urinary metabolomics identified alterations in purines, carnitines, bile acids, and steroid glucuronides associated with chronic *Opisthorchis viverrini* infection and cholangiocarcinoma^24^ ^,^ ^25^, while fecal metabolomics distinguished periductal fibrosis from cholangiocarcinoma^23^. Serum metabolomics identified spermidine, LysoPA (16:0/0:0), 12(R)-HETE, L-4-hydroxyglutamate semialdehyde, and TDCA (taurodeoxycholic acid) as discriminatory metabolites in *C. sinensis*-associated hepatocellular carcinoma^76^.

***Onchocerciasis:*** Metabolomics studies identified several candidate biomarkers in urine, plasma, and serum. An initial study reported 10 candidate biomarkers, mainly fatty acids and related metabolites^26^. N-acetyltyramine-O-β-glucuronide (NATOG) has been extensively investigated as a urinary marker of infection, disease progression, and treatment response^24^ ^,^ ^28^, although its diagnostic utility remains inconsistent. Lagatie et al.^29^ identified two plasma biomarkers associated with filarial infections. While elevated NATOG levels have been reported in mono- and co-infections with *Loa loa* and *Mansonella perstans* ^28^, other studies found limited discriminatory performance^28^ ^,^ ^31^. More recent evidence suggests that combining NATOG with additional metabolites may improve diagnostic accuracy^32^. Elevated serum serotonin, hypoxanthine, pipecolic acid, and inosine, together with reduced glycerophosphocholine, choline, and adenine, have also been reported^33^. Additionally, phosphatidylethanolamine lipid ethers detected in *O. ochengi* have been proposed as parasite-derived biomarkers^34^.

***Schistosomiasis:*** Metabolomics studies have identified metabolic alterations associated with disease progression and host-parasite interactions. Catechol estrogens distinguished uncomplicated *S. haematobium* infection from advanced bladder pathology^35^, while three bile acid-based biomarkers were proposed for chronic and advanced *S. japonicum* infection^36^. Additional studies reported alterations in lipid metabolism^35^ ^,^ ^37^, particularly involving choline and sphingolipid pathways^35^. ^1^H-NMR studies identified higher alanine, glycolaldehyde, and N-acetylglucosamines in severe periportal fibrosis and lower valine and carbohydrates in mild disease^38^. Lactate was elevated in *S. mansoni* patients coinfected with hepatitis B or C viruses, whereas HDL signals were more intense in schistosomiasis alone^39^. Additional metabolites related to gut microbiota, energy metabolism, and liver function have also been described^40^, supporting the potential of metabolomic signatures for disease staging and monitoring.

***Soil-transmitted helminthiasis:*** In *Ascaris lumbricoides*, urinary 2-methyl-pentanoyl-carnitine showed 85.7% accuracy for infection detection and 90.5% accuracy for assessing infection intensity^41^, with levels declining after albendazole treatment. For *Strongyloides stercoralis*, 28 amino acid- and lipid-related metabolites differentiated infected, uninfected, and post-treatment individuals^42^, while five candidate biomarkers achieved accuracies above 89%^43^. In *Trichuris trichiura* infection, myristic acid and DL-dipalmitoylphosphatidylcholine were strongly associated with infection intensity^89^. Overall, metabolomics may support diagnosis, treatment monitoring, and investigation of host-parasite interactions^41^ ^-^ ^43^ ^,^ ^89^.

***Taeniasis/cysticercosis:*** Neurocysticercosis (NCC) is primarily diagnosed by neuroimaging, which is often unavailable in endemic rural settings^44^. An exploratory ^1^H NMR study demonstrated moderate discrimination between NCC patients and healthy controls using urinary metabolic profiles, although the responsible metabolites were not identified^44^. Despite its exploratory nature, the study highlights the potential of non-invasive metabolomics for biomarker discovery in NCC^44^.

### Parasitic protozoan diseases

Chagas disease (CD) and African trypanosomiasis (HAT) showed convergent alterations in energy, amino acid, and lipid metabolism, including changes in aromatic and branched-chain amino acids, tricarboxylic acid cycle intermediates, acylcarnitines, and phospholipids, reflecting mitochondrial dysfunction, altered energy metabolism, and systemic inflammation. While these metabolic changes are associated with chronic cardiomyopathy in CD and central nervous system involvement in HAT, they collectively suggest shared host metabolic responses despite disease-specific biomarkers.

***Chagas disease:*** The absence of reliable biomarkers for disease progression, treatment response, and cure remains a major challenge in CD^45^. Metabolomics studies have consistently identified molecular signatures in chronic chagasic cardiomyopathy (CCC)^45^ ^-^ ^48^, including amino acids, acylcarnitines, sphingomyelins, lysophosphatidylcholines, phosphatidylcholines^45^, and metabolites of the tryptophan pathway such as serotonin, tryptamine, kynurenine, and N-acetylserotonin^48^. Integrated metabolomics identified 10-hydroxydecanoic acid and the phosphatidylethanolamines PE(18:0/20:4) and PE(18:1/20:4) as promising biomarkers for distinguishing symptomatic from asymptomatic *Trypanosoma cruzi* infection^65^. Several sphingolipids normalized after antiparasitic therapy, suggesting utility for treatment monitoring^65^. Additional studies reported alterations in tryptophan, methionine, urea, myo-inositol^46^, branched-chain amino acids, acylcarnitines, fatty acid metabolism, the tricarboxylic acid cycle, and antioxidant defenses^47^. In CCC patients, elevated progesterone and one peptide, together with reduced amino acid levels, were observed before treatment, with most metabolites normalizing after therapy^49^. Validation through targeted metabolomics and comparison with other cardiomyopathies remain necessary^45^ ^-^ ^49^ ^,^ ^65^.

***African trypanosomiasis:*** Metabolomics studies have investigated metabolic alterations associated with HAT and disease progression^50^ ^-^ ^52^. Lamour et al.^50^ reported elevated plasma levels of phenylalanine, formate, creatinine, N-acetylated glycoproteins, and triglycerides in infected individuals compared with controls. Although no stage-specific metabolic signatures were identified, increased 3-hydroxybutyrate and alanine, together with reduced mannose and urea, were associated with neurological manifestations such as somnolence and gait ataxia, suggesting potential utility as disease-stage biomarkers^51^. Using mass spectrometry, Vincent et al.^52^ identified CSF metabolites, but not urinary markers, capable of discriminating disease stages, with 100% sensitivity and specificity for distinguishing stage 1 from stage 2 disease^52^.

### Bacterial diseases

Bacterial diseases, particularly leprosy, were mainly characterized by alterations in lipid immunometabolism, including eicosanoids, resolvins, prostaglandins, and arachidonic acid pathways, highlighting the role of inflammatory resolution in disease progression. Although only one Buruli ulcer study was included, it also identified disturbances in energy and lipid metabolism, suggesting shared metabolic mechanisms among neglected mycobacterial diseases.

***Buruli ulcer:*** A single metabolomics study investigated Buruli ulcer and identified significant alterations in metabolites related to hexose metabolism, steroid hormones, acylcarnitines, purines, heme, bile acids, riboflavin, and lysolipids^53^. Altered hexose metabolism was associated with impaired glucose homeostasis, suggesting potential utility for disease monitoring^53^. 

***Leprosy:*** Metabolomics studies in leprosy have consistently highlighted alterations in lipid mediators involved in inflammatory pathways. Elevated levels of polyunsaturated fatty acids, including arachidonic acid, eicosapentaenoic acid, and docosahexaenoic acid, together with omega-3- and omega-6-derived pro-resolving mediators, were reported by Al-Mubarak et al.^54^ and Amaral et al.^87^. Additional alterations involved prostaglandin, linoleate, arachidonic acid, glycerophospholipid, and carnitine shuttle pathways^77^. Similarly, Silva et al.^55^ ^,^ ^56^ identified increased arachidonic acid, leukotriene B4, prostaglandin D2, and lipoxin A4 in household contacts who subsequently developed leprosy, proposing resolvins and one prostaglandin as potential early biomarkers^56^. Other studies reported metabolic alterations in amino acid and lipid pathways in ulcerated skin^79^ and identified urinary metabolite profiles capable of distinguishing controls from patients experiencing inflammatory reactions, suggesting potential utility for early detection of acute inflammation and prevention of tissue damage^78^.

### Viral diseases

Viral diseases, particularly dengue, were characterized by alterations in lipid metabolism, including lysophospholipids, sphingolipids, polyunsaturated fatty acids, and acylcarnitines, as well as changes in serotonin, kynurenine, and energy metabolism. These alterations were associated with inflammation, platelet activation, and disease severity, making most proposed biomarkers more suitable for prognosis than diagnosis. In contrast, rabies was predominantly associated with disruptions in cerebral energy metabolism, the tricarboxylic acid cycle, ketogenesis, and neurotransmitter pathways, consistent with central nervous system involvement.

***Dengue:*** Metabolomics studies consistently demonstrate alterations in lipid metabolism, particularly involving glycerophospholipids and sphingolipids, which are associated with disease progression and changes in lymphocyte and platelet counts^66^ ^,^ ^71^ ^,^ ^73^. Potential biomarkers include platelet-activating factor precursors, phosphatidylcholine derivatives, and triglycerides^71^, while serotonin and kynurenine have been linked to platelet aggregation and immune regulation^67^. Increased bile acids were associated with dengue hemorrhagic fever^69^, whereas comparative studies identified reduced serotonin and bile acids in dengue compared with influenza^68^. Additional metabolites related to viral replication, immune responses, disease severity, oxidative stress, and inflammation have also been reported^66^ ^-^ ^69^ ^,^ ^72^ ^,^ ^85^. Several biomarkers demonstrated promising diagnostic performance. An unidentified metabolite feature (m/z 246.2655) showed good accuracy in Ecuadorian children and adolescents^84^, while trans-cinnamic acid, L-acetylcarnitine, sphingomyelin SM(d17:1/17:0), physalactone, S-japonin, and 9-tridecynoic acid achieved good discriminatory performance in a Chinese cohort^70^. Other studies identified alterations in lipid, amino acid, and energy metabolism associated with disease progression and severe dengue^74^, as well as potential biomarkers including fatty acids, phospholipids, vitamins, amino acids, and organic acids^57^ ^,^ ^58^ ^,^ ^60^ ^,^ ^75^ ^,^ ^83^. Reduced lipoproteins, glutamine, acetate, and creatine distinguished infected patients from controls^83^, while urinary metabolomics revealed additional changes in amino acids, organic acids, and betaine^57^.

***Rabies:*** Some studies found that metabolites increased in rabies diseases using ^1^H NMR spectroscopy^61^ ^-^ ^63^. O’Sullivan *et al*.^61^ analyzed the metabolites of energy cycle, ketosis, tricarboxylic acid cycle, excitotoxic, protein catabolism, urea cycle, and lipid turnover from early, mild and late stages in cerebrospinal fluid samples. Another study showed five metabolites with higher concentration in rabies disease compared to others inflammatory diseases of the central nervous system^62^. The 3-hydroxybutyrate and acetoacetate were increased in both studies^61^ ^,^ ^62^. Additional studies using autopsy brain tissue samples reported increased levels into three different panels: brain cortex, white matter, and CSF samples compared to non-rabies patients. These metabolic changes are linked to inflammation, excitotoxicity, mitochondrial dysfunction, lactic acidosis and brain volume alterations, reflecting the progression of rabies infection^63^. From all, glutamine, lactate and taurine were elevated across two compartments (cerebral cortex and white matter)^63^.

***Snakebite envenoming:*** Only one human metabolomics study on snakebite envenoming was identified. Using untargeted and targeted metabolomics, He et al.^64^ analyzed serum samples from patients with *Naja atra* envenoming and reported significant alterations in proline, phenylalanine, and inosine compared with healthy controls. However, the study primarily emphasized findings from animal models, and the number of human participants was not clearly reported, limiting the clinical interpretation and validation of these candidate biomarkers^64^.

### Limitations

This review has several limitations. The available evidence was highly heterogeneous regarding diseases, biological matrices, metabolomics platforms, analytical methods, and reported outcomes, precluding quantitative synthesis. Most studies employed untargeted LC-MS, whereas others used GC-MS or ^1^H-NMR, limiting direct comparison of metabolite profiles across studies. Additional variability resulted from differences in sample preparation, analytical pipelines, patient populations, disease stages, and control groups. Furthermore, most studies were exploratory, involved small sample sizes, and lacked external validation. Evidence was also concentrated in a limited number of NTDs, with no human metabolomics studies identified for several WHO-defined diseases. Together, these limitations contribute to the low reproducibility of many proposed biomarkers and hinder their translation into clinical practice. Despite these challenges, this review provides a comprehensive overview of the current evidence and highlights key priorities for future biomarker discovery and validation.

## CONCLUSION

Metabolomics has emerged as a promising tool for investigating NTDs, revealing metabolic alterations associated with host-pathogen interactions, disease progression, and potential biomarkers. However, the evidence remains heterogeneous, largely exploratory, and concentrated in a few diseases, with limited validation and considerable methodological variability. Consequently, no metabolite or biomarker panel is currently ready for routine clinical use. Future research should prioritize standardized methodologies, independent validation, and greater inclusion of underrepresented diseases and endemic populations to translate metabolomic discoveries into clinically useful tools for NTD diagnosis and management.

## Data Availability

Research data is available upon request.
