# Supplementary material for: Metabolomic Biomarkers for the Human Diagnosis of Neglected Tropical Diseases: A Systematic Review
Source: Rev Soc Bras Med Trop. 2026 Aug 7;59(Suppl 1):e0298-2025. doi: 10.1590/0037-8682-0298-2025 (PMC13450828; doi:10.1590/0037-8682-0298-2025)
Supplement: Supplementary file 1 [file 1678-9849-rsbmt-59-s1-e0298-2025-md1.pdf]

Supplementary Table 1 - Search terms.

Disease	Search terms (until March 2026)
**Neglected tropical disease**	metabolomic OR metabolomics AND Neglected Tropical Disease//metabolomic OR metabolomics AND NTDs
**Buruli Ulcer**	metabolomics OR metabolomic AND buruli ulcer//metabolomics OR metabolomic AND *Mycobacterium ulcerans*
**Chagas disease**	metabolomics OR metabolomic AND Chagas disease//metabolomics OR metabolomic AND *Trypanosoma cruzi*
**Cromoblastomycose**	metabolomics OR metabolomic AND Chromoblastomycosis//metabolomics OR metabolomic AND *Cladophialophora carrionii*//metabolomics OR metabolomic AND *Fonsecaea pedrosoi*//metabolomics OR metabolomic AND *Fonsecae monophora*
**Dengue**	metabolomics OR metabolomic AND Dengue virus//metabolomics OR metabolomic AND Dengue
**Dracunculiasis (Guinea-worm disease)**	metabolomics OR metabolomic AND Dracunculisis//metabolomics OR metabolomic AND Guinea-worm disease//metabolomics OR metabolomic AND *Dracunculus medinensis*
**Echinococcosis**	metabolomics OR metabolomic AND Echinococcosis//metabolomics OR metabolomic AND *Echinococcus granulosus*//metabolomics OR metabolomic AND *Echinococcus multilocularis*//metabolomics OR metabolomic AND *Echinococcus oligarthrus*
**Foodborne trematode infections**	metabolomics OR metabolomic AND Foodborne trematode infections//metabolomics OR metabolomic AND *Clonorchis*//metabolomics OR metabolomic AND *Fasciola*//metabolomics OR metabolomic AND *Opisthorchis*//metabolomics OR metabolomic AND *Paragonimus*
**Human African Trypanosomiasis**	metabolomics OR metabolomic AND Human African Trypanosomiasis//metabolomics OR metabolomic AND *Trypanosoma brucei gambiense*//
**Leishmaniasis**	metabolomics OR metabolomic AND Leishmaniasis//metabolomics OR metabolomic AND *Leishmania*//metabolomics OR metabolomic AND *Leishmania* sp//metabolomics OR metabolomic AND *Leishmania amazonensis*//metabolomics OR metabolomic AND *Leishmania braziliensis*//metabolomics OR metabolomic AND *Leishmania infantum*//metabolomics OR metabolomic AND *Leishmania guyanensis*
**Leprosy**	metabolomics OR metabolomic AND leprosy//metabolomics OR metabolomic AND *Mycobacterium leprae*
**Lymphatic filariasis**	metabolomics OR metabolomic AND Lymphatic filariasis//metabolomics OR metabolomic AND *Wuchereria bancrofti*//metabolomics OR metabolomic AND *Brugia malayi*//metabolomics OR metabolomic AND *Brugia timori*
**Mycetoma**	metabolomics OR metabolomic AND mycetoma
**Noma**	metabolomics OR metabolomic AND noma
**Onchocerciasis**	metabolomics OR metabolomic AND onchocerciasis/metabolomics OR metabolomic AND *Onchocerca volvulus*
**Podocoaniasis**	metabolomics OR metabolomic AND Podoconiasis//metabolomics OR metabolomic AND non-filarial lymphoedema
**Rabies**	metabolomics OR metabolomic AND Rabies
**Scabies**	metabolomics OR metabolomic AND Scabies// metabolomics OR metabolomic AND *Sarcoptes scabiei var hominis*

**Schistosomiasis**	metabolomics OR metabolomic AND Schistosomiasis//metabolomics OR metabolomic AND *Schistosoma mansoni*//metabolomics OR metabolomic AND *Schistosoma japonicum*//metabolomics OR metabolomic AND *Schistosoma mekongi*//metabolomics OR metabolomic AND *Schistosoma guineensis*//metabolomics OR metabolomic AND *Schistosoma intercalatum*//metabolomics OR metabolomic AND *Schistosoma haematobium*
**Snakebite envenoming**	metabolomics OR metabolomic AND Snakebite envenoming
**Soil-transmitted helminth infections**	metabolomics OR metabolomic AND Soil-transmitted helminth//metabolomics OR metabolomic AND *Ascaris lumbricoides*//metabolomics OR metabolomic AND *Trichuris trichiura*//metabolomics OR metabolomic AND *Necator americanus*//metabolomics OR metabolomic AND *Ancylostoma duodenale*//metabolomics OR metabolomic AND *Strongyloides stercoralis*
**Sporotrichosis**	metabolomics OR metabolomic AND Sporotrichosis
**Taeniasis/cysticercosis**	metabolomics OR metabolomic AND taeniasis//metabolomics OR metabolomic AND cysticercosis/metabolomics OR metabolomic AND *Taenia solium*//metabolomics OR metabolomic AND *Taenia saginata*//metabolomics OR metabolomic AND *Taenia asiatica*
**Trachoma**	metabolomics OR metabolomic AND Trachoma//metabolomics OR metabolomic AND *Chlamydia trachomatis*
**Tungiasis**	metabolomics OR metabolomic AND Tungiasis //metabolomics OR metabolomic AND *Tunga penetrans*
**Yaws**	metabolomics OR metabolomic AND Yaws//metabolomics OR metabolomic AND *Treponema pallidum pertenue*
