# Supplementary material for: Metabolomic Biomarkers for the Human Diagnosis of Neglected Tropical Diseases: A Systematic Review
Source: Rev Soc Bras Med Trop. 2026 Aug 7;59(Suppl 1):e0298-2025. doi: 10.1590/0037-8682-0298-2025 (PMC13450828; doi:10.1590/0037-8682-0298-2025)
Supplement: Supplementary file 2 [file 1678-9849-rsbmt-59-s1-e0298-2025-md2.pdf]

Supplementary Table 2. Risk of bias for each included study. The risk of bias in each included study was assessed using a modified version of QUADOMICS, an adaptation of the Quality Assessment of Diagnostic Accuracy Studies (QUADAS) tool for studies using omic technologies. A total of six domains were assessed. Each domain was judged as having a “low”, “high”, or “unclear” risk of bias.

Study	Were selection criteria clearly described?	Was the type of sample fully described?	Were the procedures and timing of biological sample collection with respect to clinical factors described with enough detail?		Were handling and pre-analytical procedures reported in sufficient detail and similar for the whole sample?	Was the execution of the index test described in sufficient detail to permit replication of the test?	Is it likely that the presence of overfitting was avoided?	Low/high
			Clinical and physiological factors	Diagnostic and treatment procedures.				
**A01 Niang 2015**	Low risk	Low risk	High risk	High risk	High risk	Low risk	High risk	03//04
**A02 Ambrosio 2024**	Low risk	Low risk	High risk	High risk	High risk	Low risk	High risk	03//04
**A03 de Oliveira2024**	High risk	Low risk	High risk	High risk	High risk	Low risk	High risk	02//05
**A04 Díaz 2022**	High risk	Low risk	High risk	High risk	Low risk	Low risk	High risk	03//04
**A05 Gabaldón-Figueira 2024**	Low risk	Low risk	High risk	High risk	Low risk	Low risk	High risk	04//03
**A06 Golizeh 2022**	Low risk	Low risk	High risk	High risk	Low risk	Low risk	High risk	04//03
**A07 Herreros-Cabello 2024**	Low risk	Low risk	High risk	High risk	High risk	High risk	High risk	02//05
**A08 Cui 2013**	Low risk	Low risk	High risk	High risk	Low risk	Low risk	High risk	04//03
**A09 Cui 2016**	Low risk	Low risk	High risk	High risk	Low risk	Low risk	High risk	04//03
**A10 Cui 2018**	Low risk	Low risk	High risk	High risk	Low risk	Low risk	High risk	04//03
**A11 Cui 2017**	Low risk	Low risk	High risk	High risk	Low risk	Low risk	High risk	04//03
**A12 El-Bacha 2016**	Low risk	High risk	High risk	High risk	Low risk	Low risk	High risk	03//04
**A13 Fierro 2025**	High risk	Low risk	High risk	High risk	Low risk	Low risk	High risk	03//04
**A14 Josyula 2024**	High risk	Low risk	High risk	High risk	Low risk	Low risk	High risk	03//04
**A15 Liu 2026**	Low risk	Low risk	High risk	High risk	Low risk	Low risk	Low risk	05//02
**A16 Melo 2018**	Low risk	Low risk	High risk	High risk	Low risk	High risk	High risk	03//04
**A17 Rathnakumar 2023**	Low risk	Low risk	High risk	High risk	Low risk	Low risk	High risk	04//03
**A18 Shahfiza 2017**	High risk	Low risk	High risk	High risk	Low risk	Low risk	High risk	03//04
**A19 Shahfiza 2015**	High risk	Low risk	High risk	High risk	Low risk	Low risk	High risk	03//04
**A20 Shahfiza 2015**	High risk	Low risk	High risk	High risk	Low risk	Low risk	High risk	03//04
**A21 Shrinet 2016**	Low risk	Low risk	High risk	High risk	Low risk	Low risk	High risk	04//03
**A22 Soe 2020**	High risk	Low risk	High risk	High risk	High risk	Low risk	High risk	02//05
**A23 Soma 2025**	Low risk	Low risk	High risk	High risk	High risk	Low risk	High risk	03//04
**A24 Voge 2016**	Low risk	Low risk	High risk	High risk	Low risk	Low risk	High risk	04//03
**A25 Aimaiti 2025**	Low risk	Low risk	High risk	High risk	Low risk	High risk	High risk	03//04
**A26 Bai 2023**	Low risk	Low risk	High risk	High risk	High risk	Low risk	High risk	03//04
**A27 Ciftci 2021**	Low risk	High risk	High risk	High risk	High risk	Low risk	High risk	02//05
**A28 Lin 2019**	Low risk	Low risk	High risk	High risk	Low risk	Low risk	High risk	04//03
**A29 Raj 2024**	Low risk	Low risk	High risk	High risk	Low risk	High risk	High risk	03//04
**A30 Tuerxun 2025**	High risk	Low risk	High risk	High risk	Low risk	Low risk	High risk	03//04
**A31 Alsaleh 2019**	Low risk	Low risk	High risk	High risk	Low risk	High risk	High risk	03//04
**A32 Alsaleh 2023**	Low risk	Low risk	High risk	High risk	Low risk	High risk	High risk	03//04
**A33 Hua 2026**	Low risk	Low risk	High risk	High risk	Low risk	High risk	High risk	03//04

**A34 Tang 2025**	Low risk	Low risk	High risk	Low risk	High risk	Low risk	High risk	04//03
**A35 Treeriya 2023**	Low risk	Low risk	High risk	High risk	Low risk	Low risk	High risk	04//03
**A36 Zhang 2023**	Low risk	Low risk	High risk	High risk	Low risk	Low risk	High risk	04//03
**A37 Lamour 2017**	Low risk	Low risk	High risk	High risk	Low risk	Low risk	High risk	04//03
**A38 Lamour 2015**	Low risk	High risk	High risk	High risk	Low risk	Low risk	High risk	03//04
**A39 Vincent 2016**	Low risk	Low risk	High risk	High risk	Low risk	Low risk	High risk	04//03
**A40 Al-Mubarak 2011**	Low risk	Low risk	High risk	High risk	Low risk	Low risk	High risk	04//03
**A41 Amaral 2013**	Low risk	Low risk	High risk	High risk	High risk	Low risk	High risk	03//04
**A42 Fairley 2024**	Low risk	High risk	High risk	High risk	Low risk	High risk	High risk	02//05
**A43 Mayboroda 2016**	High risk	Low risk	High risk	High risk	Low risk	Low risk	High risk	03//04
**A44 Silva 2023**	Low risk	Low risk	High risk	High risk	Low risk	Low risk	High risk	04//03
**A45 Silva 2017**	Low risk	Low risk	High risk	High risk	High risk	Low risk	High risk	03//04
**A46 Wang 2023**	Low risk	Low risk	High risk	High risk	Low risk	High risk	High risk	03//04
**A47 Bennuru 2017**	High risk	High risk	High risk	High risk	High risk	Low risk	High risk	01//06
**A48 Denery 2010**	High risk	High risk	High risk	High risk	High risk	Low risk	High risk	01//06
**A49 Globisch 2017**	High risk	Low risk	High risk	High risk	High risk	Low risk	High risk	02//05
**A50 Globisch 2013**	High risk	Low risk	High risk	High risk	High risk	Low risk	High risk	02//05
**A51 Lagatie 2016**	Low risk	Low risk	High risk	High risk	High risk	Low risk	High risk	03//04
**A52 Lagatie 2021**	Low risk	High risk	High risk	High risk	Low risk	Low risk	Low risk	04//03
**A53 Wewer 2021**	Low risk	Low risk	High risk	High risk	Low risk	Low risk	High risk	04//03
**A54 Wewer 2017**	High risk	High risk	High risk	High risk	High risk	Low risk	High risk	01//06
**A55 Hotterbeekx 2020**	Low risk	Low risk	High risk	High risk	High risk	High risk	High risk	02//05
**A56 French 2018**	Low risk	Low risk	High risk	High risk	Low risk	Low risk	High risk	04//03
**A57 O'Sullivan 2013**	Low risk	Low risk	High risk	High risk	Low risk	Low risk	High risk	04//03
**A58 Reinke 2013**	Low risk	Low risk	High risk	High risk	High risk	High risk	High risk	02//05
**A59 Adebayo 2018**	Low risk	High risk	High risk	High risk	Low risk	Low risk	High risk	03//04
**A60 Balog 2011**	High risk	Low risk	High risk	High risk	Low risk	Low risk	High risk	03//04
**A61 Fialho 2025**	Low risk	Low risk	High risk	High risk	High risk	Low risk	High risk	03//04
**A62 Gouveia 2017**	Low risk	Low risk	High risk	High risk	Low risk	Low risk	High risk	04//03
**A63 Li 2024**	Low risk	Low risk	High risk	High risk	Low risk	Low risk	High risk	04//03
**A64 Osakunor 2020**	Low risk	Low risk	High risk	High risk	Low risk	Low risk	High risk	04//03
**A65 Rodrigues 2022**	Low risk	Low risk	High risk	High risk	Low risk	Low risk	High risk	04//03
**A66 Tawana-Ndolo 2023**	Low risk	Low risk	High risk	High risk	Low risk	Low risk	High risk	04//03
**A67 Walusimbi 2026**	Low risk	Low risk	High risk	High risk	High risk	Low risk	High risk	03//04
**A68 Zhou 2023**	Low risk	Low risk	High risk	High risk	Low risk	Low risk	High risk	04//03
**A69 He 2023**	High risk	Low risk	High risk	High risk	High risk	Low risk	High risk	02//05
**A70 Jenkins 2018**	Low risk	Low risk	High risk	High risk	Low risk	Low risk	High risk	04//03
**A71 Lagatie 2020**	Low risk	High risk	High risk	High risk	Low risk	Low risk	Low risk	04//03
**A72 Montanhaur 2022**	High risk	Low risk	High risk	High risk	Low risk	Low risk	High risk	03//04
**A73 Zhang 2025**	Low risk	Low risk	High risk	High risk	Low risk	High risk	High risk	03//04
**A74 Lopez 2022**	Low risk	Low risk	High risk	High risk	Low risk	Low risk	High risk	04//03
